# A Systematic Review of Randomized Controlled Trials on Virtual Reality Application in Pediatric Patients

**DOI:** 10.7759/cureus.30543

**Published:** 2022-10-21

**Authors:** Ashish Varma, Waqar M Naqvi, Salima Mulla, Samana Syed, Sumit Thakur, Sakshi P Arora, Anuj R Varma, Smruti Besekar

**Affiliations:** 1 Pediatrics, Jawaharlal Nehru Medical College, Datta Meghe Institute of Medical Sciences, Wardha, IND; 2 Physical Medicine and Rehabilitation, Ravi Nair Physiotherapy College, Datta Meghe Institute of Medical Sciences, Wardha, IND; 3 Research, NKP Salve Institute of Medical Sciences and Research Center, Nagpur, IND; 4 Physical Medicine and Rehabilitation, SDM College of Physiotherapy, Dharwad, IND; 5 Cardiorespiratory Physiotherapy, Krishnai Hospital, Mumbai, IND; 6 Pediatrics, David Ferguson Neonatal Unit, Newport, GBR; 7 Community Health Physiotherapy, Ravi Nair Physiotherapy College, Datta Meghe Institute of Medical Sciences, Wardha, IND; 8 Medicine, Jawaharlal Nehru Medical College, Datta Meghe Institute of Medical Sciences, Wardha, IND; 9 Research, Humen Edutech, Nagpur, IND

**Keywords:** pediatric, vr, systematic review, virtual reality, pain, cerebral palsy

## Abstract

Virtual reality is a novel approach for distracting and alleviating anxiety, pain, and other complications during medical procedures, and it can be more effective than conventional methods. In virtual reality, the patient is completely immersed in the virtual environment, which is used to make patients feel more comfortable and can provide a positive prognosis. The data were searched by using the Boolean operator “AND” between the search phrases “Virtual reality,” and “Pediatrics” and the relevant literature was extracted. The inclusion criteria were the free full text, randomized controlled trials, studies between 2016 and 2022 and pediatric patients. This systematic review was conducted to compare randomized controlled trials of virtual reality applications in pediatric patients in different clinical settings. Of the included 15 randomized controlled trials, 12 studies were on pain and anxiety, two on brain injury and cerebral palsy, and one on awareness among asthmatic patients. This review concluded that virtual reality exposure has a beneficial effect on pediatric patients in reducing pain and anxiety, improving muscle strength and dexterity, and awareness among asthmatic patients.

## Introduction and background

Pain, anxiety, and stress are generally observed during medical procedures in pediatric patients with poor prognosis, nervousness, loss of appetite, sleep disturbances, the effort to escape, and post-traumatic stress manifestations. Moreover, negligence toward health is a result of altogether pain, anxiety, and stress. Hence, new therapies are crucial to alleviate pain and anxiety in children and can be achieved by distraction during clinical procedures [1].

Distraction methods such as the use of music, movies, and toys have been shown to have a positive impact on decreasing pain and anxiety. Virtual reality (VR) is the newest technique to provide distraction during medical procedures and has been proven to be more efficacious than other conventional methods, and is usually made up of a computer-generated environment, and three-dimensional use and orientation are feasible [2]. The user views this environment through advanced head-mounted displays (HMDs), which feature a vivid range of sight and a movement trailing system. The perception of living in VR was due to total immersion which promotes less pain during the procedure using greater immersion and less attention to pain perception. VR is specially created for children, as they are more attracted to imaginative games and play [1,2].

VR is usually divided into non-immersive, fully immersive, semi-immersive, and augmented VR which is widely used in clinical practice for distraction and to keep patients calm [3]. Hence, this review was conducted to highlight the VR applications in pediatric patients with different clinical symptoms.

## Review

Research methodology

**Figure 1:**
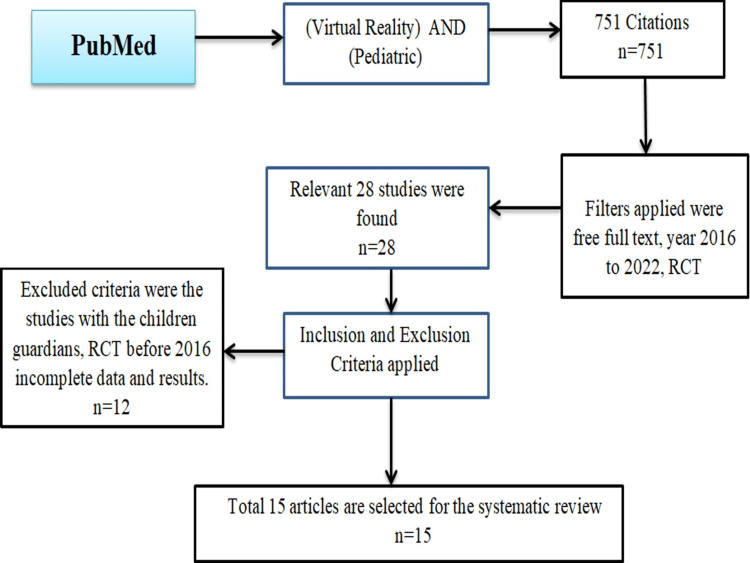
RCT selection method and inclusion criteria. RCT = Randomized Controlled Trial, n = Number of RCTs

For the purpose of performing this systematic review, the PRISMA-S statement for reporting systematic reviews was utilized [4]. Randomized Controlled Trials (RCTs) that were released between 2016 and July 2022 were looked for in PubMed and Google Scholar. Using the Boolean operator “AND,” the search phrases from the two search themes were merged to search the databases. The use of the text phrases “virtual reality” and “pediatrics,” aimed to quantify VR exposure (VRE) in pediatric patients. This review included English-language studies that examined the applications of VR distractions and improvements among pediatric patients and evaluated the impact of VR on pain management, nervousness, neurorehabilitation, and dental surgeries. The articles enlisted, were with the title “virtual reality” AND “pediatrics” AND “children,” free full abstract, and, randomized controlled trials. The peer review, observational studies, case reports, trials that were before 2016, trial data and results that were not declared or incomplete trials, and studies of children’s parents, nursing staff, and adults were excluded. Figure 1 shows the RCTs selection method and inclusion criteria.

Results

Only 15 RCTs were enrolled in this study from the PubMed database. Various variables related to VR applications were investigated and enlisted in this systematic review. Meta-analysis was not feasible due to differences in setting, inclusion criteria, and outcome characterization and assessment. However, comparisons among similar studies provide a guide for VR use in pediatric patients and relevant practices in real-life settings. Information regarding the author's name, names of journals, place of study, and year of publication were mentioned in Table 1.

**Table 1:** The information about the RCTs used for comparison. BMC = BioMed Central, RCTs = Randomized Controlled Trials

Sr No	Authors Name	Name of Journal	Place of study	Year of publication
1	Eijlers et al. [1]	European Journals of Anesthesiology	Netherlands	2018
2	Jung et al. [3]	International Anesthesia Research Society	San Francisco	2019
3	Felemban et al. [5]	BMC oral health	Saudi Arabia	2019
4	Choi et al. [6]	Developmental medicine and child neurology	Seoul	2020
5	El Shamy et al. [7]	Journal of Musculoskeletal Research	Saudi Arabia	2017
6	Xiang et al. [8]	Jama Network	United States	2019.
7	Jeffery et al. [9]	Jama Network	United States	2019
8	Chelsea et al. [10]	Journal Medical Internet research	United States	2022
9	Hundert et al. [11]	The clinical journal of pain	Canada	2021
10	Hoag et al. [12]	The journal of medical internet research	Wisconsin.	2019,
11	Du et al. [13]	Brain and behavior	China.	2020
12	Kim [14]	Journal of Educational Evaluation for Health Professionals	Korea	2021
13	Aztori [15]	Journal of Educational Evaluation and Public Health	Italy	2018,
14	Choo et al. [16]	The Tohoku Journal of experimental medicine	South Korea	2016
15	Lee et al. [17]	PloS One	Seoul	2019

Table 2 shows the comparison between sample size, inclusion criteria, purpose, and outcomes of the RCTs.

**Table 2:** The comparison among the RCT, their purpose and outcome. FLACC = Face, Legs, Activity, Cry, Consolability, PIVC = Peripheral Intravenous Catheter, SPM = Standard Preparatory Manual, VR-MRI = Virtual Reality Magnetic Resonance Imaging, VR = Virtual Reality, GI = Guided Imagery, HMDs = Head Mounted Displays, VRTT = Virtual Reality Treadmill Training, IV = Intravenous, RCT = Randomized Controlled Trial, CG = Control Group.

Sr no	Authors	Sample size	Inclusion	Aims and Objectives	Outcome
1	Eijlers et al. [1]	200 VRE= 100 CAU=100	4-12 years of children for an elective day of surgery under General anesthesia	- assessed anxiety and pain during induction of anesthesia. -determined self-reported pain and anxiety, surfacing delirium, required analgesia – morphine (Scale used for anxiety assessment was Modified Yale Preoperative Anxiety Scale)-	There was no significant dissimilarity between virtual reality exposure (VRE) and care as usual (CAU) in reducing anxiety, pain or delirium or parental anxiety during elective surgery. Anyhow, after VRE, less requirement of analgesia after painful surgery
2	Jung et al. [3]	71 VR=34 No VR=37	Age 5-12 years - Under general anesthesia for elective surgery.	- assessed anxiety in patient undergoing elective surgery using Modified Yale Preoperative Anxiety Scale. - To determine post induction parental anxiety, measured by State Trial anxiety Inventory Pediatric Induction Compliance Parental satisfaction	-The application of immersive audio-visual distraction with VR leads a decrease anxiety in pediatric patients receiving general anesthesia (GA). -Inducing anesthesia in children with perioperative VR was also a successful, non-invasive method of providing anxiolytics.
3	Felemban et al. [5]	50 VR=25 CG=25	-6-12 years of children -needed buccal infiltration anesthesia	Determined patient anxiety; heart rate and for pain -Face, legs activity, cry, consolability (FLACC) behavioral pain assessment scale and Wong Bakers faces.	When used on pediatric patients, virtual reality goggles had an equivalent effect to screen distraction during buccal infiltration anesthesia on heart rate and discomfort Despite the type of distraction used, female subjects, children, and after receiving local anesthetics, patients with increased heart rates at baselines were more likely to report experiencing more pain.
4	Choi JY et al. [6]	80 VR= 40 CG=38	3-16 years of age with brain injury such as cerebral palsy	assessed the effectiveness of a wearable multi-inertial sensor virtual reality rehabilitation system to enhance upper-extremity function in children with brain injury.	the severe motor impairments showed noticeable enhancement as compared to the less severe impairment in children with brain damage
5	El Shamy et al. [7]	40 Conventional Physiotherapy group=20 VR=20	-5-8 years of age - Obstetric Brachial Plexus injury the form of Erb’s type (C5, C6) injuries obtained from medical records.	-determined smartphone VR game use among pediatric patients with burns on dressing pain.	The virtual reality program significantly surpasses conventional physiotherapy in terms of enhancing upper arm functions in children with obstetric brachial plexus injury.
6	Xiang et al. [8]	90 Active VR= 31 Passive VR=20 Control group= 29	- 6-17 years – patient in outpatient clinic -level I pediatric trauma center -English Speaking	- assessed pain reduction in burn patients	In comparison to those in the control and standard care groups as well as those in the passive VR and control groups, Participants in the active VR group scored significantly higher for overall pain and lower for the severe pain.
7	Jeffery et al. [9]	107 Conventional group=54 VR=53	- Children of 10 to 21 years of age -For PIVC insertion in two medical settings (a radiology department and an infusion center)	- Patient’s pain (measured by the Faces Pain Scale–Revised) - Following PIVC placement, anxiety was expressed by the patient, their caregiver, and the clinician (assessed using a visual analogue scale).	When compared to patients who got conventional treatment, patients PIVC placement patients who got a VR intervention disclosed that pain and anxiety were significantly reduced.
8	Chelsea et al. [10]	92 VR-MRI= 30 SPM=24 CLP= 30	Aged 4-13 years patients' caregivers.	- Determined anxiety reported by child and caregiver.	No clinically relevant differences in the outcome were seen in the intervention and control group in reducing anxiety in children. Caregiver were satisfied with the virtual reality magnetic resonance imaging (VR-MRI) app as compared to standard preparatory manual (SPM)
9	Hundert et al. [11]	40 VR=20 CG=20	-Children and adolescents - aged 8 to 18 years -speaking English, -cancer therapy	Evaluated VR's early therapy active distraction control (iPad) on anxiety and distress	When compared to iPad distraction, VR may lessen anxiety and distress.
10	Hoag et al. [12]	50 GI =26 VR= 24	-8 to 25 years -Children and young adults were enrolled from the department like hematology, oncology, and blood and marrow transplant clinics at a children's hospital	Assessed the effectiveness of guided imagery (GI) and virtual reality (VR) on procedural pain and state anxiety in children and young adults undergoing non-sedated procedures.	Patients with chronic pain had shown efficacious response to VR and for the anxiety GI is useful solution
11	Du et al. [13]	86 VR=42, CG=41	Children’s who were need for deciduous teeth extraction under influence of local anesthesia	-to assess the effects of a virtual reality (VR) intervention on young patients' anxiety of the dentist, their perception of pain, and their behavior, as well as the likelihood that they may experience simulator sickness during local anesthetic and primary tooth extraction	Children's dental fear and pain perception can be considerably reduced by using VR headgear during local anesthetic and primary tooth extraction without experiencing simulator sickness. Although, it was not adequate and useful technique for children with extreme anxiety
12	Kim [14]	30 VR=15 CG=15	-Children aged 9 to 13 years -Sensitive to aerosol allergen. -Asthma patient	Evaluated the awareness and learning among asthmatic children about environmental control by use of immersive virtual reality (VR) technology .	The outcome suggested that there was no significant difference in the use of VR technology among both groups. The outcome of environmental management awareness, memory, and intent to act obtained before, just after, and four weeks after instruction comparing participants in VR education wearing HMDs and those using printed materials did not significantly differ from each other.
13	Aztori [15]	82 VR=41 No VR=41	7-17 years age group -venipuncture with distinct kidney diseases ---known of Italian language.	- Evaluated VR analgesic effectiveness in pediatric and adolescent renal disease patients undergoing venipuncture Estimation of worst pain of the venipuncture, using a 0–10 Verbal Numeric Rating Scale	This study showed the beneficial applications of immersive virtual reality (VR) distraction for pain relief and control as a psychological remedy during venipuncture in patients with chronic renal disease. During venipuncture, VR markedly boosted fun while greatly reducing the worst pain and pain unpleasantness
14	Choo et al. [16]	18 VRTT=9 TT=9	aged 4-16 years; Patients with cerebral palsy	- assessed the effectiveness of VR treadmill exercise on gait, equilibrium, muscular strength, and dexterity in pediatric patients with cerebral palsy.	After training, gait performance is improved by VRTT. The treadmill gait training with virtual reality is particularly helpful at enhancing children with spastic cerebral palsy's ability to balance, which is the secondary goal
15	Lee et al. [17]	19 VR=9 CG=10	-aged 2 to 6 years - patients for IV placement or needle insertion procedure	Examined the viability and possible effectiveness of employing a dome screen and a virtual reality (VR) environment to divert children in midst of intravenous (IV) placement.	A preliminary study revealed that virtual reality on a dome screen might be an effective way to divert young children from a needle procedure.

All study designs were interventional and consistent with the inclusion criteria for this review. All studies used a randomized controlled design to demonstrate the effectiveness of VR in several parameters in pediatric patients. The majority of studies (n=12) evaluated patients' experiences with venipuncture, peripheral intravenous catheter (PIVC) installation, dental extraction, elective surgery under general anesthesia, and pain and anxiety related to those procedures. Two RCTs (n=2) assessed dexterity and muscular power in patients with cerebral palsy (CP) and one RCT (n=1) demonstrated the use of VR for creating awareness among asthmatic patients.

Discussion

This novel systematic analysis emphasizes randomized controlled research that details VR technology applications. The current medical uses of VR technology are pain distraction, anxiety relief, and rehabilitation, as revealed by data from 15 studies that were eligible for this review.

Procedure-related pain that is not properly handled is extremely upsetting and can increase pain sensitivity over time, decreasing the probability that an adult would seek medical attention and even fewer possibilities in pediatric patients [13]. VR is an experimentally established, practical, and economical intervention to alleviate pain and anxiety during regular venipuncture operations in the pediatric setting. Interventions using VR can help patients feel more satisfied with their care, caregivers, and overall health by reducing needle anxiety, traumatic reactions to medical procedures, and adverse reactions [10].

The goal of the previously conducted RCT was to identify less risky and non-pharmacological alternatives to treat pain and discomfort caused by repeated treatments when sedation is unnecessary. VRE has not, however, demonstrated any appreciable differences and patients frequently required the use of pharmaceutical treatments and analgesics (morphine), hence the use of immersive VR such as headset and audiovisual distraction led to a reduction in anxiety in the operating room before elective surgery, as the study was a pragmatic implementation, and it used established outcome scale that was validated and showed less observer bias [3].

Another RCT reported that VR did not significantly reduce pain or anxiety in patients undergoing elective surgery. A single evaluation performed before entering the recovery room did not provide a thorough understanding of the postoperative effects of VRE. The study was discontinued in 21 children by removing the headset and was heavy and larger in size, which might have caused discomfort to the patients, as the majority of subjects were 4-5 years of age, and minor procedures were included [1]. Compared to traditional ways of managing behavior in the field of medical and dental sciences, VR distraction is beneficial in reducing pain and anxiety. According to a study by Gold et al., VR decreased anxiety and acute procedural pain during phlebotomy procedures [2].

Chances of dentist phobia, non-cooperative conduct at the time of treatment, and the potential to avoid future dental appointments have all been linked to unpleasant prior dental experiences, including the use of local anesthetics [18]. The Heart Rate (HR) was used to illustrate anxiety as a physiological measurement, and it showed significant elevations after screen use when compared to the control group, as well as increased mean HR levels during the administration of the local anesthetic and its procedures. The study investigated pain during anesthesia utilization, which may have created fear in children and may have contributed to the elevated level of HR in the test group. There was inadequate blinding of the investigator, no direct observation, and increased inconsistency due to the administration of local anesthesia by different clinicians for the assessment of the Face, Legs, Activity, Cry, Consolability (FLACC) Behavioral Pain Assessment Scale [5].

Similarly, the positive influence of VR on pain, distress, needle phobia, and treatment in patients who routinely undergo PIVC procedures irrespective of their chronic status has been demonstrated [9]. According to Hundert et al. findings, children and adolescents utilized VR as an intervention and desired to use VR for subsequent Subcutaneous Port (SCP) insertions in cancer patients. Due to the limited sample size, wide confidence intervals, and dichotomized results, the findings were described conservatively [11].

Another RCT reported that VR using a dome screen is a practical means of diverting young children during intravenous (IV) insertion; however, its efficacy could not be validated because of the limited sample size [12]. Similar to this study, the use of VR headgear during local anesthesia and the removal of deciduous teeth can considerably reduce children's fear of the dentist and their perception of pain without the development of simulator sickness [12], which is similar to the findings of Lahti et al. [19]. In another study, most participants concluded that the procedures were associated with less discomfort, and some groups of patients reported high levels of pain across interventions. Children with a history of chronic pain and perception of the predisposing pain responded more favorably to VR. The limitations of this study include its heterogeneous sample and the absence of intervention [12]. Aztori et al. conducted RCT on children with chronic kidney disease, it involved the use of an immersive VR venipuncture on a psychological pain management strategy during venipuncture, and throughout this process, VR considerably increased fun while significantly reducing the greatest pain and pain unpleasantness. The children who participated in the study were younger than eight years; therefore, they were unable to discern between different pain levels, fear was not examined, and only one venipuncture was used to test pain [15].

There is not much research that determined the VR distraction outcome on anxiety, pain, gender, and age respectively. Similarly, another study examined the effects of audio distraction visually on anxiety in children aged four to six years compared to those aged six to eight years and discovered that it effectively reduced anxiety in the treatment groups [20]. Hence, in this systematic review, it was unclear, whether VR applications significantly decreased pain and anxiety.

A major objective for pediatric patients with CP is independent walking, which was covered in this review on the use of VR in children with brain injuries and subjected to clinical and community-focused rehabilitation [21]. This study examined how children with CP walked, balanced themselves, developed their muscles, and used their gross motor skills after treadmill training [16]. Since the children were able to walk without the aid of walkers after using VR treadmill training, it was employed without partial body weight support. This improved muscular strength is evidenced by the children's increased walking speed and lower limb weight-bearing capacity. The VR Treadmill Training (VRTT) group's walking activities greatly improved as a result of the transfer of VR walking training skills to actual walking surroundings. However, given that the intervention lasted for only eight weeks, the results may not necessarily indicate possible long-term therapeutic success [16]. According to Schlough et al. children with CP who combine aerobic exercise with treadmill gait training has noticeably increased muscle strength [21].

Similarly, VR games effectively motivate children with CP and, as a result, represent a therapeutic approach that can be utilized to speed up rehabilitation training [22]. The execution of daily tasks, dexterity, and active forearm supination motion improved more effectively with VR training. Children with more severe motor deficits benefit considerably from VR training [23]. By providing implicit learning, tangible activities, and concentrated attention, VR-based rehabilitation improves the motor learning processes. Choi et al. used game content for training purposes, such as the performance of daily living activities such as eating, pouring water, and cooking [6]. A fundamental concept in rehabilitation is task-specific training, which focuses on repeatedly performing certain functional tasks. For task-specific training, it has been recommended that tasks be repetitive, pertinent to the patient, randomly allocated, and reinforced with a positive review [6,24].

It is possible to use therapeutic techniques like task-directed exercise for VR rehabilitation. Thus, more engagement in daily activities, as determined by the Pediatric Evaluation of Disability Inventory Computer Adaptive Test (PEDI-CAT) in the study, may have resulted from improvements in upper limb function [24]. Another study concluded that VR might be challenging to show improvements in the quality of CP therapy. For instance, spastic quadriplegia and diplegia patients between the ages of eight and 12 years were treated for eight weeks for one and a half hours each week of treatment was given [24,25]. Similarly, Chen et al. examined how VR could help four children with CP between the ages of four and eight years to meet their activity goals. Additionally, it is critical to use home-based treatment strategies to extend the duration of interventions and boost the effectiveness of VR intervention [23].

The establishment and comparison of awareness and education among asthma patients showed an efficacious impact of immersive VR content. Additionally, the asthma control status of the experimental and control groups was compared and was informative for the participants [14]. The positive side of this review, it has included the generalizability of the study, because of the use of diverse inclusion criteria, and also involved cases from dentistry, oncology, surgeries, hematology, physiotherapy, and neurology. The only drawback of this systematic review is that, although relatively few articles were included and several parameters were investigated simultaneously, the overall utilization of VR cannot be examined and understood in general. Some studies have demonstrated the advantages of alternative therapies over conventional therapies, and vice versa. The adverse effects among pediatrics using VR were not included in the systematic review and could not provide a definitive conclusion that VR therapy is superior to standard care or traditional practices.

## Conclusions

In this study, the use of VRE among pediatric patients showed a beneficial impact, and in some RCTs, it did not cause any significant difference when compared with the usual care. Gamification and VR applications can be helpful for pediatric patients. However, there is currently little empirical data and poor evidence. Future research can be conducted by including more than two databases and including more searching terms with combination and permutation for accessing and analyzing more relevant literature.
